# Effect of different impression techniques on marginal integrity of CAD-CAM milled all-on-four mandibular frameworks: an in vitro study

**DOI:** 10.1186/s12903-025-05784-y

**Published:** 2025-04-07

**Authors:** Reem A. Mahmoud, Ahmed A. Abdel Hakim, Nermeen A. Rady

**Affiliations:** Department of Prosthodontics, Faculty of Dentistry, Alexandria University, Champollion St., Azarita, Alexandria, 21527 Egypt

**Keywords:** All-on-four, CAD-CAM milled frameworks, VSXE impression material, Intraoral scanner

## Abstract

**Background:**

To guarantee a passive fit, full arch implant supported prostheses require scrupulous impressions. The accuracy of conventional and digital impressions is still up for debate, despite several studies comparing both acquisition techniques. The present study aimed to compare mandibular full arch implant impressions by assessing the vertical misfit of implant supported frameworks obtained through conventional and digital impressions.

**Methods:**

To simulate the “All-on-4” scenario, a completely edentulous epoxy mandibular reference model was prepared with the installment of two straight implants in the anterior region and two 30-degree angled implants in the posterior region. Two acquisition techniques were evaluated: the conventional impression technique (CI group, *n* = 11) with open tray splinted impression copings using vinyl siloxane ether (VSXE) impression and the digital impression technique (DI group, *n* = 11) using Medit i-700 intraoral scanner (IOS). To create virtual models, the Medit T-Series laboratory scanner was used to scan the models created by the CI group. Scans obtained from both groups were saved as STL files for framework design. Screw retained bars (*n* = 22) were designed on the virtual models and then machined in cobalt chromium. The frameworks fabricated using both impressions were screwed to the reference model, evaluated using the Sheffield test, and the vertical misfits were analyzed under a stereomicroscope at 80× magnification. Comparisons between the two study groups were performed using independent samples t-test, and the average vertical misfits of each multi-unit abutment in each group were compared by using the ANOVA test followed by a Post Hoc test (adjusted Bonferroni) for pairwise comparison. At *P* <.05, statistical significance was assessed.

**Results:**

When tightening the screw at multi-unit abutment #45, the vertical misfits of the frameworks manufactured by DI group (82.34 ± 5.05 μm) were lower than those of the CI group (91.09 ± 6.29 μm) with significant difference at *P* =.002, while no statistical significant difference was reported in the average vertical misfit between the CI group (43.60 ± 11.93 μm) and the DI group (43.90 ± 5.31 μm) (*P* =.940) while securing the screw at multi-unit abutment #35.

**Conclusions:**

Achieving a passive fit for implant supported frameworks in completely edentulous patients is quite challenging. A fully digital workflow offers a steadfast alternative to conventional methods with vertical misfits that differ based on the impression technique, though these differences are typically not statistically significant.

## Background

Successful rehabilitation of fully edentulous patients with implant supported restorations necessitates a high level of accuracy, as today’s patients anticipate longevity, function, and aesthetics from their definitive restorations [1]. Maló et al. produced clinical documentation of the “All-on-4” concept of edentulous mandible rehabilitation in 2003. This concept was introduced to minimize the constraints of fixed restorations for posterior regions with reduced bone quality and quantity [2, 3]. The synchronous and equal contact at the implant framework interface following screw tightening is referred to as the passive fit [4] and this passivity is essential for maintaining osseointegration and preventing stress building at the bone-implant interface [5].

For an implant supported prosthesis to fit passively, a precise impression is indispensable. The direct “open-tray” method and the indirect “closed-tray” method are examples of conventional implant impression procedures. Most studies have indicated that greater accuracy is achieved with splinted impressions compared to non-splinted ones and with open and individualized trays rather than closed trays [6–8].

The new generation vinyl siloxane ether (VSXE) impression development integrates the advantages of both polyvinyl siloxane and polyether materials and is specifically designed for monophase impressions. The VSXE impression material is vigorous as it secures transfer due to exceptionally high final hardness (shore-A 65), ensuring the reliable fixation of implant posts. VSXE also possesses excellent flowability providing optimal coverage of transfer posts with extended working time and quick setting time. However, there is a lack of sufficient scientific evidence to demonstrate its clinical effectiveness as an impression material for the full arch implant supported prostheses.

Digital technology has made significant strides in dentistry not long ago, with a wide range of restorations that are now created using many computer-aided design and computer-aided manufacturing (CAD-CAM) systems. Digital scanners enable clinicians to generate virtual images of intraoral structures either in the dental laboratory or directly in the oral cavity [9].

However, scanning completely edentulous arches remains a challenge [10]. In some studies, difficulties with full arch digital implant scanning were mentioned, including greater inter-implant distances and edentulous segments lacking in geometric structure resulting in inaccuracies in image superimposition and differentiation between identical implant scan bodies [11–13].

Despite these limitations, other researchers demonstrated promising results for intraoral scanning in the rehabilitation of the full arch implant [14–16]. Therefore, there is still disagreement over the routine use of digital impressions to create complete arch implant supported prostheses, even with the noteworthy advancements in recent research.

The purpose of this in vitro study was to compare different acquisition techniques for full arch implant supported prostheses by evaluating the conventional impression technique with VSXE against the digital impression technique on a reference model simulating the All-on-4 situation. The study focused on measuring the vertical misfit at the abutment prosthesis interface. The null hypothesis was that no difference would be found in the vertical misfit between CAD-CAM milled frameworks designed from models obtained through various acquisition techniques.

## Methods

**Fig. 1:**
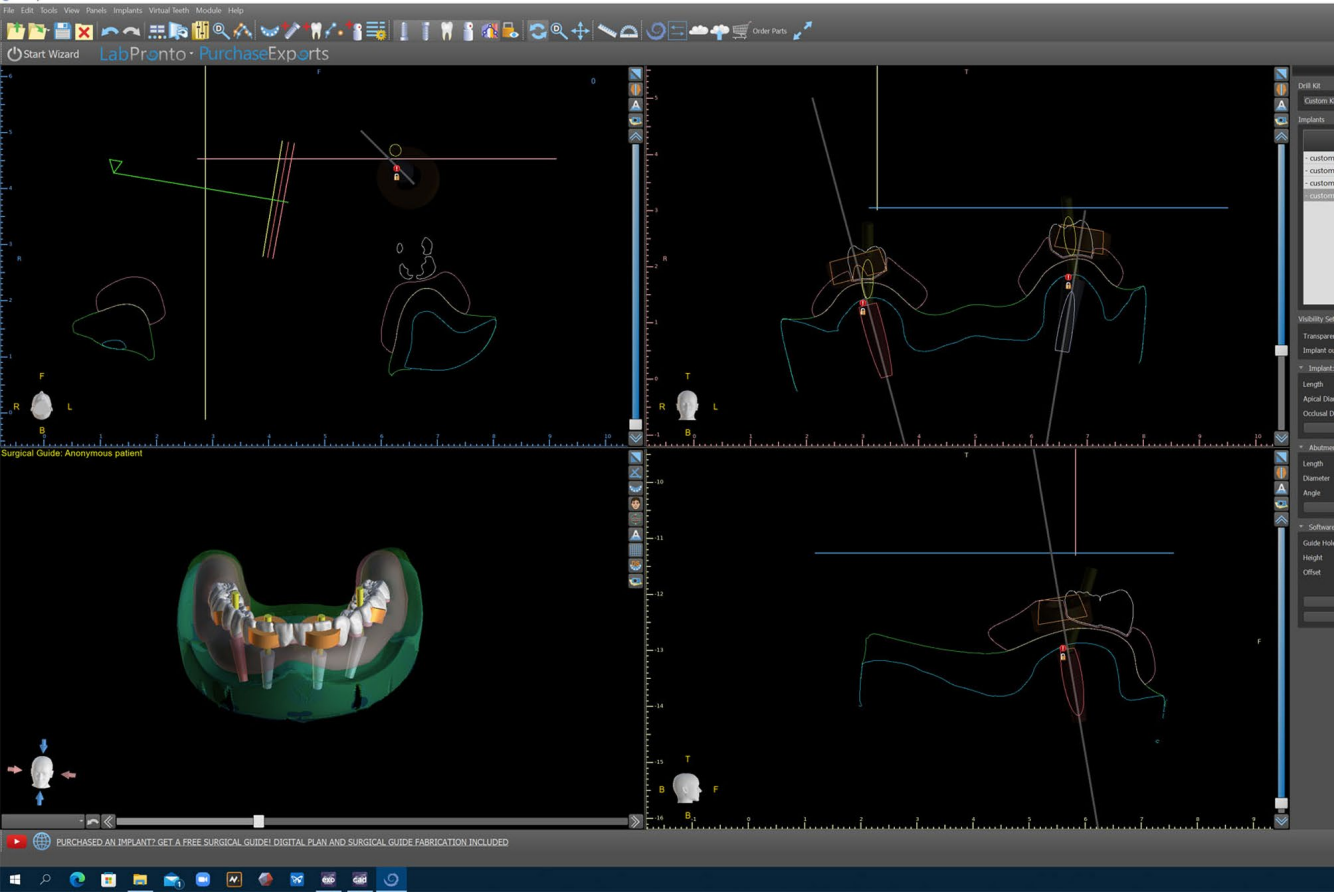
Surgical guide design using Exocad and Blue Sky Plan software

The reference model, which depicted a fully edentulous mandibular arch, was a prefabricated epoxy model covered in pink gingiva. Prosthetically driven implant locations and angulations were designed using Exocad (DentalCad 2016, Exocad, Darmstadt, Germany) and Blue Sky Plan software, to replicate an “All-on-4” clinical scenario (Fig. 1). Accordingly, the surgical guide was designed and 3D printed using the Formlabs Form 2^®^ 3D printer utilizing resin as the printing material.

Four implants (VITRONEX Implant system, Italy) were installed in the epoxy model in the interforaminal region where two parallel implants (diameter: 4.2 mm; length: 11.5 mm) placed anteriorly at the sites of mandibular lateral incisors and two 30-degrees posterior implants (diameter: 4.2 mm; length: 13 mm) placed at the sites of mandibular second premolars. A torque of 30 Ncm was used to screw four multi-unit abutments (MUAs) (VITRONEX Implant system, Italy) over the implants. Two of which were straight MUAs (FLW-UMD3-MPI, straight MUA abutment, VITRONEX Implant system, Italy) screwed on the anterior implants, while the other two MUAs (FLW-UMA305-MPI, angled 17° MUA abutment, VITRONEX Implant system, Italy) were angled and screwed on the posterior implants.

Subsequently, two types of impressions were assessed: (i) CI using VSXE (VSXE^®^ ONE monophase, Kattenbach GmbH & Co. KG., Germany) (ii) DI using IOS (Medit i-700 wireless, MEDIT Corp, Seoul, Republic of Korea).

The sample size was estimated based on assuming a 5% alpha error and 80% study power. The mean (SD) marginal gap of CAD/CAM milled frameworks using vinyl polyether siloxane impression material and intraoral scanners was 48.59 (4.16) µm and 54.985 (5.59) µm [17]. The sample size was calculated based on comparing two independent means using pooled SD = 4.875. The minimum sample size was calculated to be 10 samples per group, increased to 11 samples to make up for processing errors. There were 22 samples in the study, 11 in each group. The sample size was calculated based on Rosner’s method [18] calculated by Brant’s sample size calculator at the University of British Columbia [19].

**Fig. 2:**
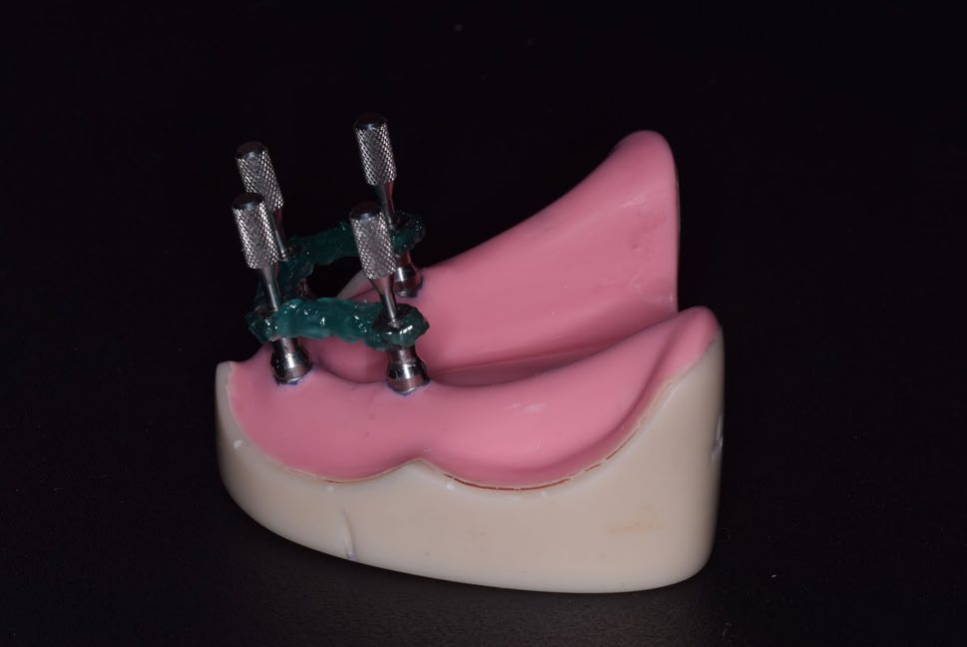
Impression copings screwed to the MUAs and splinted together with low-shrinkage pattern resin

**Fig. 3:**
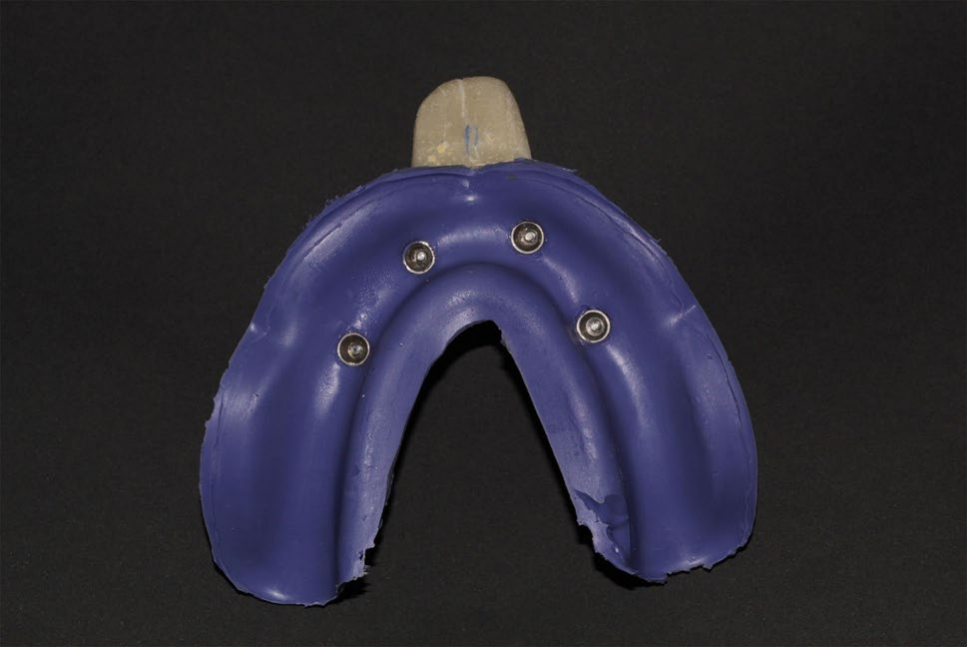
Conventional open tray impressions using VSXE

For CI, impression copings (FLU-TR-MPE, MUA impression coping, VITRONEX Implant system, Italy) were screwed (15 Ncm) to the MUAs and splinted together using low-shrinkage pattern resin (Pattern Resin LS; GC, Tokyo, Japan) (Fig. 2), subsequently sectioned with carborundum disc, and reassembled to mitigate the resin polymerization shrinkage. Eleven conventional open tray impressions were made with VSXE, where a thin coat of VSXE adhesive (Identium^®^ Adhesive, Kettenbach GmbH & Co. KG., Germany) was applied onto the dry impression tray and left to dry for 5 min. Then, the impression material was extruded in both the custom made and around the transfer copings, and the impression tray was placed over the reference model. Five minutes later, the transfer copings were unscrewed, and the impression tray was gently removed from the reference model (Fig. 3).

Afterward, MUA analogs (FLU-AN-MPE, MUA lab analog, VITRONEX Implant system, Italy) were screwed to the transfer copings, then type IV stone (CAM-Stone N; SILADENT, Goslar, Germany) was mixed per the manufacturer’s directions and poured over the impression. The casts were then trimmed and finished. After 1 h, the transfer copings were unscrewed, and the impression trays were removed from the poured stone models. Eleven stone models were produced by repeating this procedure 11 times.

**Fig. 4:**
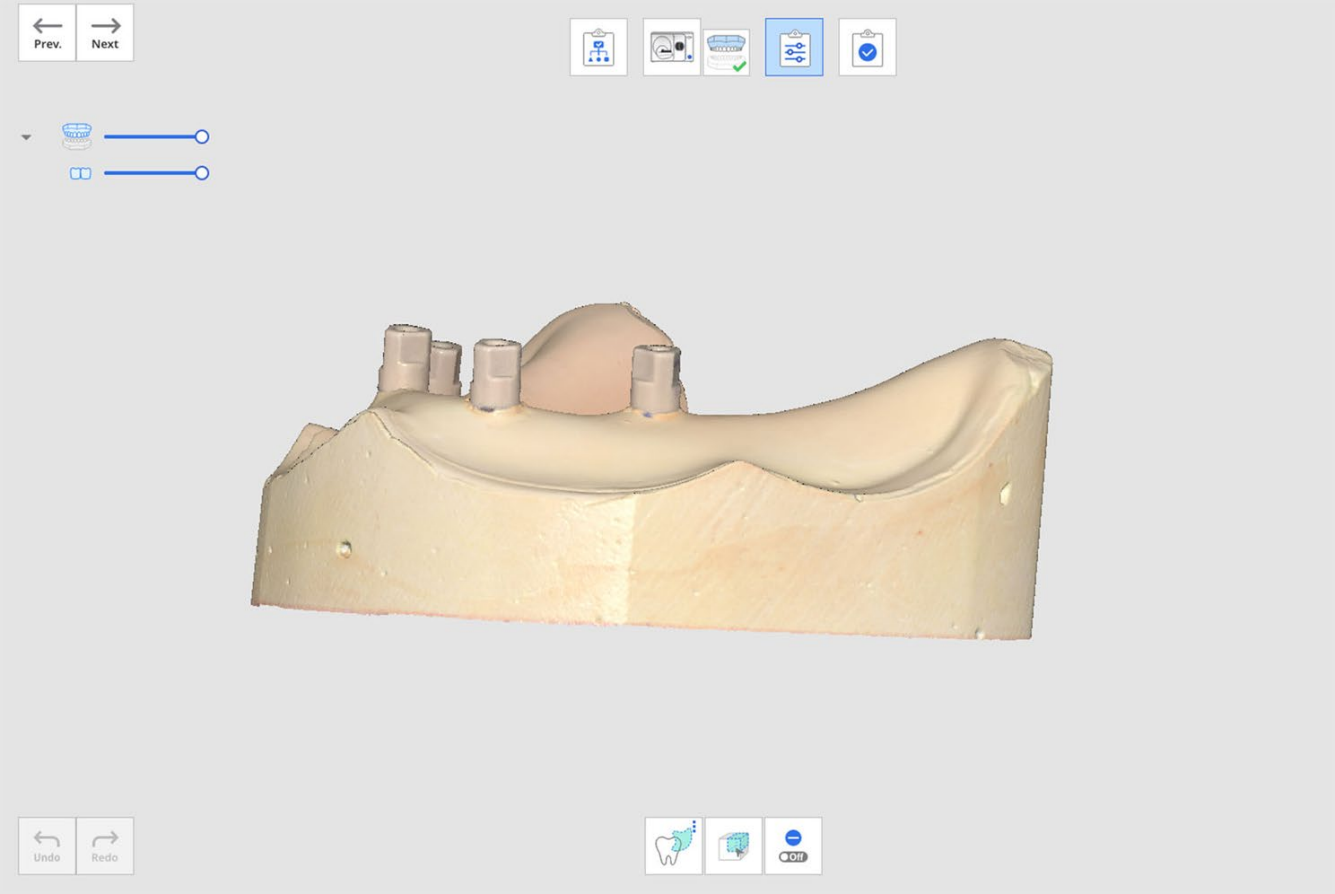
Scan of multi-unit scan body screwed onto the multi-unit analogs of the stone models using Medit T-710 laboratory scanner

Scan bodies (Multi-unit scan body, NeoBiotech Implant System, Seoul, Republic of Korea) (diameter: 4.8 mm) were screwed (15 Ncm) onto the MUA analogs of the 11 stone models then scanned using Medit T-710 laboratory scanner (MEDIT Corp, Seoul, Republic of Korea), producing 11 virtual models that were named and saved as STL files for framework design (Fig. 4).

**Fig. 5:**
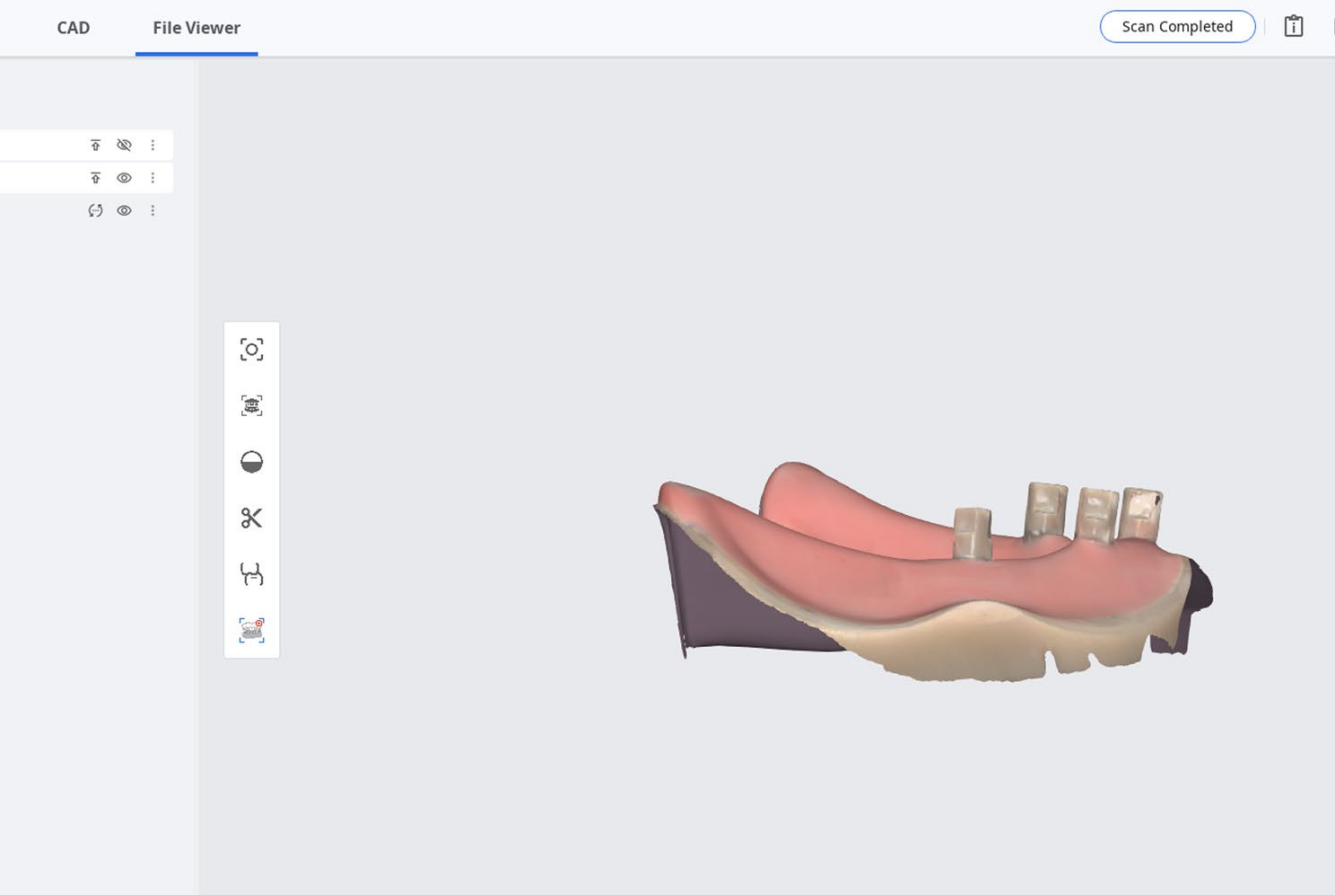
Scan of multi-unit scan body screwed onto the multi-unit analogs of the reference model using Medit i-700 intraoral scanner

For DI, scan bodies (multi-unit scan body, NeoBiotech Implant System, Seoul, Republic of Korea) (diameter: 4.8 mm) were screwed (15 Ncm) onto the MUAs on the reference model before the digital impression. The reference model was scanned using IOS (Medit i-700 wireless, MEDIT Corp, Seoul, Republic of Korea). The scanning methods applied by the operator were suggested by the manufacturers. To prevent needless reflections, the operator aimed to capture all the details of each intraoral scan body without asserting too much on them from the same angle. Eleven digital impressions were made to create 11 virtual models, which were then named and saved as STL files for framework design (Fig. 5).

**Fig. 6:**
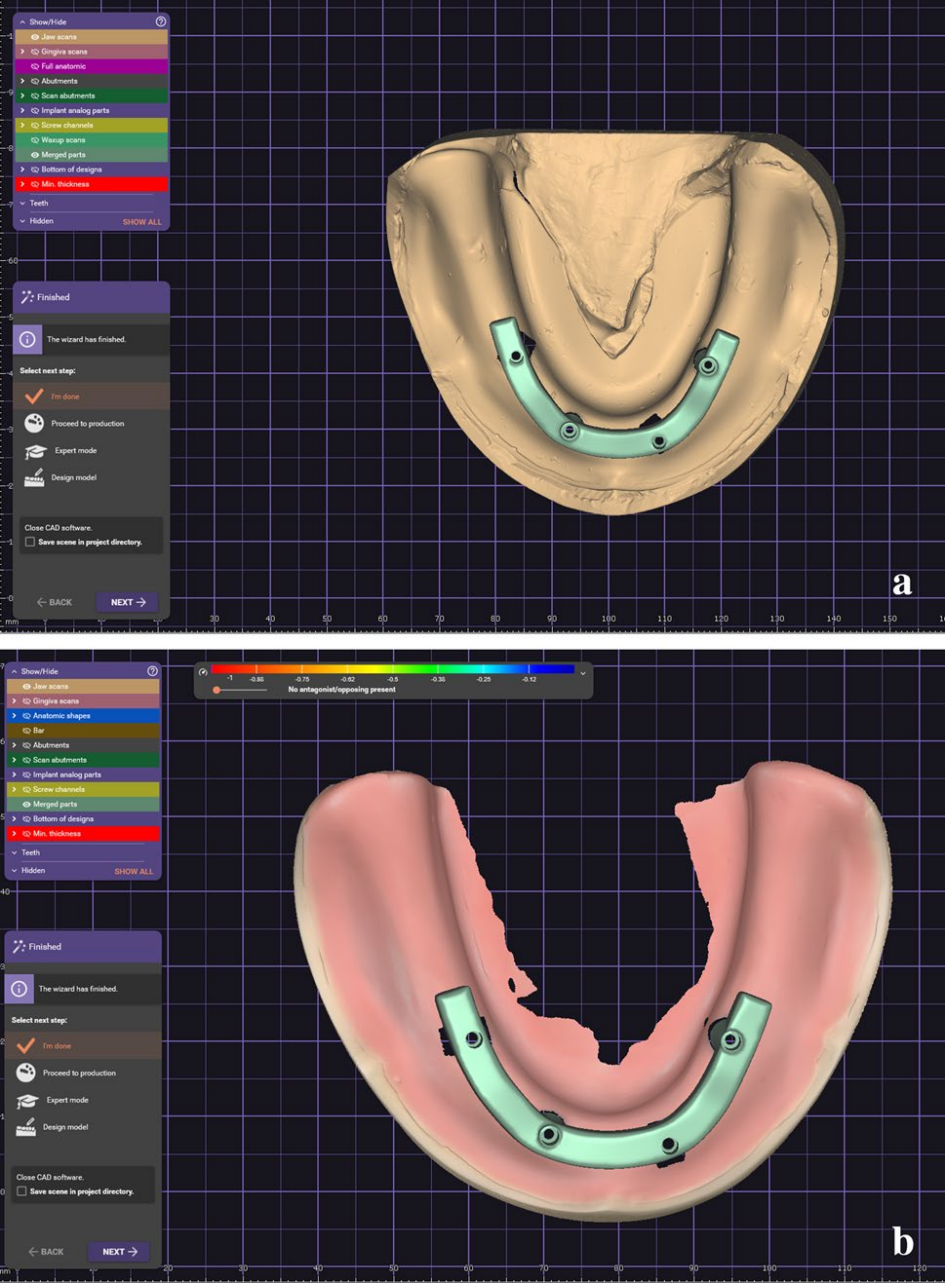
(**a**) Design of screw-retained prosthetic framework bar type for the CI group, (**b**) Design of screw-retained prosthetic framework bar type for the DI group

Each of the STL files generated by the virtual models from the CI group and those produced by the DI group were imported into the CAD software. A screw-retained prosthetic framework of bar type was designed, and the framework’s external anatomy served as the reference for each design of the subsequent models. Conclusively, 11 bars from the CI group (Fig. 6A) and 11 from the DI group (Fig. 6B) were obtained, named, and saved as STL files.

Each file was transmitted to a 5-axis milling machine (EDX5 EMAR Dental mills, Egypt) where cobalt chromium discs (Arum Cr-Co disc Magnum Splendidum, GmbH, Germany) (diameter: 98.5 mm; height: 16 mm) were milled into a bar framework. None of the frameworks were adjusted nor finished in any way. Only the sprues attached to the frameworks were cut and smoothed using a bur.

Each cobalt chromium bar was placed onto the MUAs of the reference model. Then, the Sheffield test (one-screw test) was applied for all frameworks, by tightening only one screw at the terminal end of one abutment and evaluating the fit of the remaining framework to the abutment platform on the opposite side. On the left side, using a calibrated hand wrench, each framework was secured on the reference model with only one screw at the most distal MUA screwed with a torque of 15 Ncm.

The vertical misfit, commonly observed as a gap, on the surface of the MUA shoulder and respective framework platform on the opposite side, was evaluated at 80× magnification in a frontal view both labially/buccally and lingually under the stereo microscope (Olympus SZ 11, Japan). The same procedure was repeated from the right side.

**Fig. 7:**
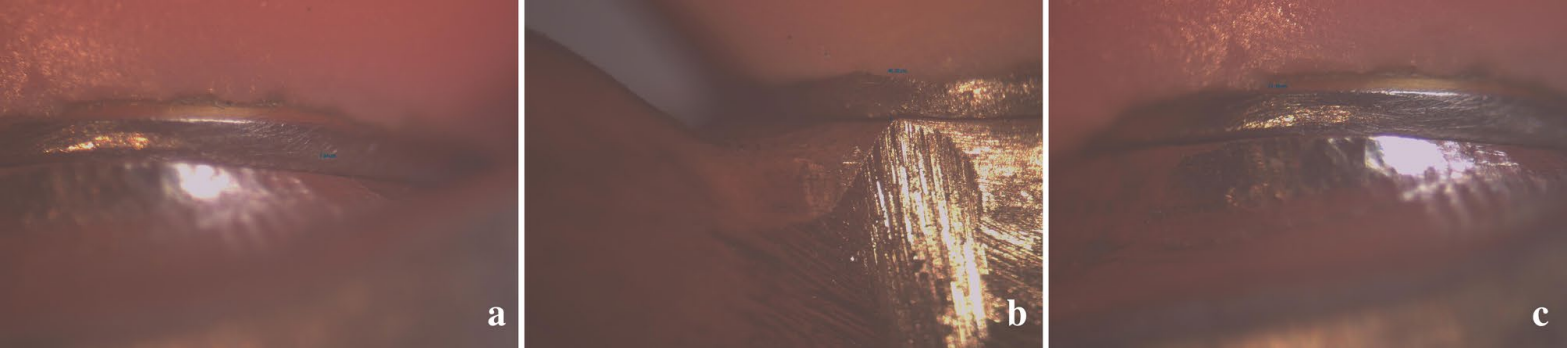
(**a**) Marginal gap measurements using a stereomicroscope (Labially), (**b**) Marginal gap measurements using a stereomicroscope (Buccally), (**c**) Marginal gap measurements using a stereomicroscope (Lingually)

From the stereomicroscope-generated images, vertical measurements at each line angle were captured on the edge of the labial/buccal (Fig. 7A, B) and lingual (Fig. 7C) surface of the gap between the MUA shoulder and respective framework platform. The mean of these 4 measurements was considered the vertical misfit of each MUA and the mean of the 4 misfit values of the 4 MUAs’ altogether was considered the mean value of each bar misfit. The mean of the misfit values of the 11 bars was considered the final mean value of each group, where each bar was submitted to 2 different measurements (with 1 screw tightened on each side) in each group respectively.

Normality was tested using the Shapiro-Wilk normality test. Variables were normally distributed. So, parametric statistics were adopted. Descriptive statistics were calculated as means, (SD), Standard error of the mean (SEM), 95% Confidence Interval (CI), and 25th Percentile– 75th Percentile. Comparisons between the two study groups were performed using independent samples t-test with calculation of mean differences and 95% CI of the difference, and the average vertical misfits of each multi-unit abutment in each group were compared by using the One Way Analysis Of Variance ANOVA test followed by a Post Hoc test (adjusted Bonferroni) for pairwise comparison. Statistical significance was tested at *P* <.05. Data were analyzed using IBM SPSS for Windows (Version 27.0, IBM Corp.).

## Results

The vertical misfit of the CAD-CAM milled frameworks made by different acquisition techniques was assessed in 2 ways, first by tightening only one screw at the most distal MUA (MUA #35) on the left side and then by the same token on the right side (MUA #45).

### Average vertical misfit (µm) in the CI and the DI groups where MUA #35 was screwed

**Fig. 8:**
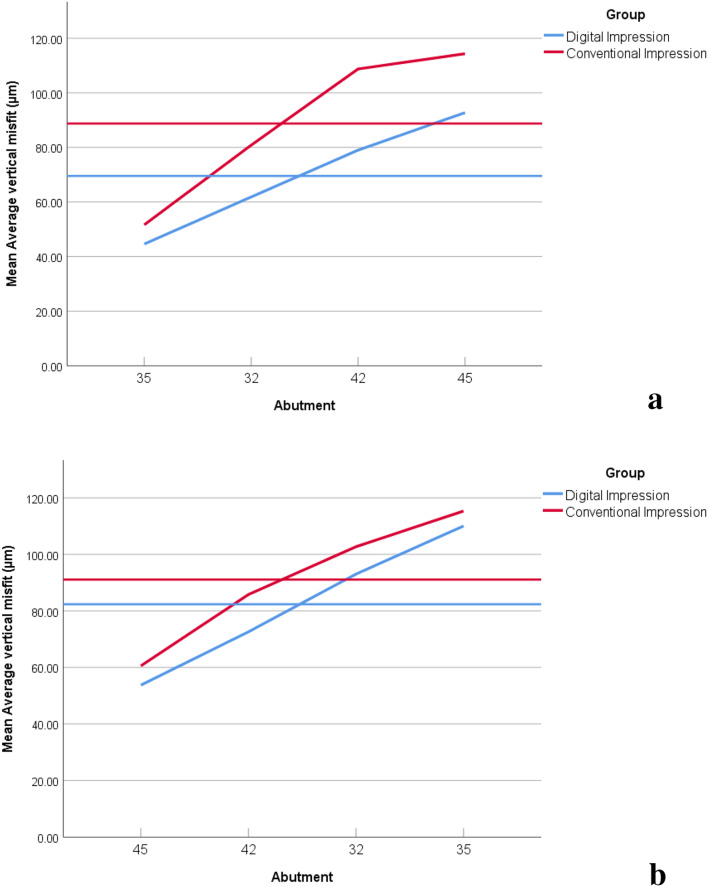
(**a**) Stereomicroscope readings of average vertical misfit (µm) in the two groups (multi-unit abutment #35 screwed), (**b**) Stereomicroscope readings of average vertical misfit (µm) in the two groups (multi-unit abutment #45 screwed)

At #35, there was no statistically significant difference in the average vertical misfit (µm) between the CI group (51.63 ± 16.80) and the DI group (44.59 ± 2.92) (*P* =.199). At #32, it was statistically significantly higher in the CI group (80.82 ± 6.20) than in the DI group (61.83 ± 12.71) (*P* <.001). At #42, it was statistically significantly higher in the CI group (108.75 ± 12.64) than in the DI group (79.01 ± 6.87) (*P* <.001). At #45, it was statistically significantly higher in the CI group (114.33 ± 15.91) than in the DI group (92.74 ± 15.46) (*P* =.004). The pairwise intragroup comparison revealed a statistically significant difference in the average vertical misfit in the CI group (F = 49.893, *P* <.001) and the DI group (F = 42.119, *P <*.001) among different MUAs. Details of pairwise comparisons are illustrated in Table 1, (Fig. 8A).

**Table 1:** Stereomicroscope readings of average vertical misfit (µm) in the two groups (MUA #35 screwed)

Average vertical misfit (µm) (All Surfaces)	Group		Mean Difference (95% CI)	Test of significance *p*-value
	Conventional Impression (*n* = 11)	Digital Impression (*n* = 11)		
At #35				
- Min.– Max.	23.83–82.47	37.42–48.13		t_(W)(df=10.602)_ = 1.369*P* = 0.199 NS
- Mean ± SD	51.63^a^ ± 16.80	44.59^a^ ± 2.92	7.037	
- SEM	5.06	0.88	(15.403–4.3291)	
- 95% CI of the mean	40.34–62.91	42.63–46.55		
- 25th Percentile– 75th Percentile	35.22–60.54	43.71–46.87		
**At #32**				
- Min.– Max.	71.14–95.18	41.80-93.44		t_(df=20)_ = 4.454 *P <*.001*
- Mean ± SD	80.82^b^ ± 6.20	61.83^b^ ± 12.71	18.989	
- SEM	1.87	3.83	(27.882–10.096)	
- 95% CI of the mean	76.65–84.98	53.29–70.37		
- 25th Percentile– 75th Percentile	78.21–84.06	56.58–66.48		
**At #42**				
- Min.– Max.	84.48-137.01	62.25–85.70		t_(df=20)_ = 6.856 *P <*.001*
- Mean ± SD	108.75^c, d^±12.64	79.01^c^ ± 6.87	29.742	
- SEM	3.81	2.07	(38.791–20.693)	
- 95% CI of the mean	100.26-117.24	74.39–83.62		
- 25th Percentile– 75th Percentile	104.42-112.82	75.73–83.74		
**At #45**				
- Min.– Max.	92.28-139.97	59.30-120.94		t_(df=20)_ = 3.228 *P =*.004*
- Mean ± SD	114.33^c, d^±15.91	92.74^d^ ± 15.46	21.592	
- SEM	4.80	4.66	(35.543–7.641)	
- 95% CI of the mean	103.64-125.02	82.35-103.12		
- 25th Percentile– 75th Percentile	98.19-122.62	86.53-105.04		
**Test of significance** ***P-*** **value**	F_(df=3)_ = 49.893 *P* <.001*	F_(df=3)_ = 42.119 *P* <.001*		
**Post-Hoc Comparison**				
- At #35 vs. At #32	*P <*.001*	*P* =.003*		
- At #35 vs. At #42	*P <*.001*	*P <*.001*		
- At #35 vs. At #45	*P <*.001*	*P <*.001*		
- At #32 vs. At #42	*P <*.001*	*P* =.003*		
- At #32 vs. At #45	*P <*.001*	*P <*.001*		
- At #42 vs. At #45	*P* =.769 NS	*P* =.022*		

n: Number of bars Min-Max: Minimum– Maximum

SD.: Standard Deviation CI: Confidence interval

SEM: Standard Error for the mean MW: Mann-Whitney U

F: F of One Way Analysis Of Variance (ANOVA) df = degree of freedom

Post-hoc comparison using the Tukey HSD method *Statistically significant (*P* <.05)

NA: Statistically not significant (*P* ≥.05)

### Average vertical misfit (µm) in the CI and the DI groups where MUA #45 was screwed

At #45, the average vertical misfit (µm) was statistically significantly higher in the CI group (60.56 ± 4.93) than in the DI group (53.73 ± 8.80) (*P* =.036). At #42, it was statistically significantly higher in the CI group (85.79 ± 9.99) than in the DI group (72.59 ± 11.22) (*P* =.009). At #32, there was no statistically significant difference between the CI group (102.71 ± 14.38) and the DI group (93.02 ± 17.85) (*P* =.176). At #35, there was no statistically significant difference between the CI group (115.32 ± 11.43) and the DI group (110.04 ± 10.26) (*P* =.268). The pairwise intragroup comparison revealed a statistically significant difference in the average vertical misfit in the CI group (F = 53.471, *P* <.001) and the DI group (F = 41.976, *P <*.001) among different MUAs. Details of pairwise comparisons are illustrated in Table 2, (Fig. 8B).

**Table 2:** Stereomicroscope readings of average vertical misfit (µm) in the two groups (MUA #45 screwed)

Average vertical misfit (µm)	Group		Mean Difference (95% CI)	Test of significance *p*-value
	Conventional Impression (*n* = 11)	Digital Impression (*n* = 11)		
At #45				
- Min.– Max.	53.06–69.45	40.37–70.12		t_(df=20)_ = 2.245 *P =*.036*
- Mean ± SD	60.56^d^ ± 4.93	53.73^d^ ± 8.80	6.831	
- SEM	1.49	2.65	(13.177–0.485)	
- 95% CI of the mean	57.25–63.87	47.82–59.65		
- 25th Percentile– 75th Percentile	57.08–62.37	47.05–62.54		
**At #42**				
- Min.– Max.	67.65-105.26	60.38–98.38		t_(df=20)_ = 2.914 *P =*.009*
- Mean ± SD	85.79^c^ ± 9.99	72.59^c^ ± 11.22		
- SEM	3.01	3.38	13.198	
- 95% CI of the mean	79.08–92.50	65.06–80.13	(22.645–3.751)	
25th Percentile– 75th Percentile	80.92–90.33	64.09–80.26		
**At #32**				
- Min.– Max.	82.69-126.51	50.96–120.40		
- Mean ± SD	102.71^b^ ± 14.38	93.02^b^ ± 17.85	9.689	
- SEM	4.34	5.38	(24.103–4.725)	t_(df=20)_ = 1.402*P =* 0.176 NS
- 95% CI of the mean	93.05-112.37	81.03-105.01		
25th Percentile– 75th Percentile	90.00-112.38	85.10-99.03		
**At #35**				
- Min.– Max.	95.19-127.48	87.65-127.76		t_(df=20)_ = 1.140*P =* 0.268 NS
- Mean ± SD	115.32^a^ ± 11.43	110.04^a^ ± 10.26	5.279	
- SEM	3.45	3.09	(14.939–4.380)	
- 95% CI of the mean 25th Percentile– 75th Percentile	107.64–123.00 105.00-123.62	103.15-116.93 103.34-115.79		
**Test of significance** ***p-*** **value**	F_(BF)(df=3, 30.265)_ = 53.471 *P* <.001*	F_(df=3)_ = 41.976 *P* <.001*		
**Post-Hoc Comparison**				
- At #45 vs. At #42	*P <*.001*	*P* =.006*		
- At #45 vs. At #32	*P <*.001*	*P <*.001*		
- At #45 vs. At #35	*P <*.001*	*P <*.001*		
- At #42 vs. At #32	*P* =.004*	*P* =.002*		
- At #42 vs. At #35	*P <*.001*	*P <*.001*		
- At #32 vs. At #35	*P* =.042*	*P* =.014*		

n: Number of bars Min-Max: Minimum– Maximum

SD.: Standard Deviation CI: Confidence interval

SEM: Standard Error for the mean MW: Mann-Whitney U

F: F of One Way Analysis Of Variance (ANOVA) Post-hoc comparison using the Tukey HSD method

df = degree of freedom *Statistically significant (*P* <.05)

NA: Statistically not significant (*P* ≥.05)

### Overall average vertical misfit (µm) in the CI and the DI groups

At #35, there was no statistically significant difference in the average vertical misfit (µm) between the CI group (43.60 ± 11.93) and the DI group (43.90 ± 5.31) (*P* =.940). At #45, it was statistically significantly higher in the CI group (91.09 ± 6.29) than the DI group (82.34 ± 5.05) (*P* =.002) illustrated in Table 3.

**Table 3:** Stereomicroscope readings of average vertical misfit (µm) in the two groups (Overall)

Average vertical misfit (µm)	Group		Mean Difference (95% CI)	Test of significance *p*-value
	Conventional Impression (*n* = 11)	Digital Impression (*n* = 11)		
At #35				
- Min.– Max.	25.71–58.91	35.72–52.59		t_(W)(df=13.814)_ = 0.077 *P =*.940 NS
- Mean ± SD	43.60 ± 11.93	43.90 ± 5.31	0.301	
- SEM	3.60	1.60	(8.152–8.755)	
- 95% CI of the mean	35.59–51.61	40.33–47.47		
- 25th Percentile– 75th Percentile	31.26–56.02	39.03–48.13		
**At #45**				
- Min.– Max.	82.77-100.61	74.49–90.86		t_(df=20)_ = 3.595 *P =*.002*
- Mean ± SD	91.09 ± 6.29	82.34 ± 5.05	8.749	
- SEM	1.90	1.52	(13.826–3.673)	
- 95% CI of the mean	86.87–95.32	78.95–85.74		
- 25th Percentile– 75th Percentile	85.09–96.24	78.67–84.19		

n: Number of bars Min-Max: Minimum– Maximum

SD.: Standard Deviation CI: Confidence interval

SEM: Standard Error for the mean W: Welch’s t-test

*Statistically significant (*P* <.05) NS: Statistically not significant (*P* ≥.05)

## Discussion

Consistent with the results, the null hypothesis was rejected due to significant differences in vertical misfit between screw retained milled frameworks designed from virtual models obtained using different impression techniques. The DI group exhibited significantly lower misfit readings compared to the CI group.

The All-on-4 treatment concept was designed to address the needs of edentulous patients with anatomical constraints, helping to eliminate the need for additional surgical procedures in those with limited bone quantity. It also enables the placement of a fixed prosthesis in a single surgery, particularly benefiting elderly patients with systemic health concerns. The success of all‑on‑4 treatment concept restorations relies heavily on attaining a passive fit [3]. The quality of fit for implant supported prostheses is crucial for ensuring long-term success. Achieving a perfect “passive fit” in screw retained prostheses is unattainable, as frameworks always undergo some degree of deformation, even under optimal fit conditions. Impressions with insufficient accuracy cannot accurately replicate the actual implant position, leading to misfits between the framework and the underlying abutments. This misfit can result in pathological concerns, which have been extensively documented in the literature [20].

Different acquisition techniques were used in this study; conventional impression technique using VSXE impression, poured then scanned using Medit T-Series laboratory scanner, and digital impression technique using Medit i-700 IOS. This IOS was selected due to its availability in our faculty department. Additionally, at the time of our study, it was one of the latest models on the market. Furthermore, a previous study on precision identified the Medit i700 as the most accurate device [3]. The conventional technique includes clinical and laboratory steps which might result in minute inaccuracies [15]. So in the present study, standardization of many variables was achieved by using custom trays (designed for having repeatable uniform impression thickness), direct impression technique, laboratory pouring procedures, splinting material and its thickness, operator effect (the use of a single examiner), and scanning of poured casts. Employing digital scanning for full arch implant prostheses seems to present one of the most defying clinical scenarios [13, 14]. Therefore, with a larger area to digitize, more images are required, which results in more image stitching and an increased likelihood of errors [21]. Mandibular and tongue movements, along with excessive salivation and the operator’s skill level are additional factors that complicate the process [14].

A new set of burs was employed to mill each bar in this study to reduce frequent errors in the bar margins and remove any potential impact of milling bur efficiency on the resulting misfit. All the frameworks were placed directly on the MUAs, as the use of Ti-bases between the framework and MUAs was avoided due to the manual setting and luting process, which could potentially lead to misfit [22]. It is important to emphasize that all bars were screwed onto the same reference model. To minimize the risk of micro deformations in the mandibular reference model, each bar was screwed onto the reference model only once.

Dimensional analysis through direct measurement of vertical gaps is a valid approach for assessing the quality of techniques used to fabricate implant supported prostheses [23]. The current investigation utilized a stereomicroscope because it provided clear, high-resolution images that made it possible to precisely delineate the measurement lines. Acquiring vertical misfit values in micrometers provides a clinically relevant understanding, as it allows for observation of the actual misfit values in the prosthetic structure [24]. Consequently, this enables comparisons with misfit levels documented in the literature that are deemed acceptable. The maximum acceptable misfit value has no consensus. However, many studies and reviews considered misfit values up to 150–200 μm are considered clinically acceptable [11, 15]. The study’s results, derived from CI and DI groups, showed a degree of adaptability that fell within the ranges the literature deems adequate: CI (MUA#35 screwed) = 43.60 ± 11.93 μm, CI (MUA #45 screwed) = 91.09 ± 6.29 μm, DI (MUA #35 screwed) = 43.90 ± 5.31 μm, DI (MUA #45 screwed) = 82.34 ± 5.05 μm. It can be inferred that both impression techniques produced excellent prostheses, even though the misfit values obtained in this in vitro study cannot be directly translated to clinical settings.

An in vitro study comparing full arch implant supported prostheses reported a mean marginal discrepancy between the conventional technique and the digital technique (135.1 μm vs.63.14 μm, respectively), as measured by optical microscopy with digital technique having preeminence over the conventional technique [15]. What is more, Menini et al. [25] compared various impression techniques for multiple implants, where the Sheffield test revealed a mean gap of 0.022 ± 0.023 mm (the best TI), 0.063 ± 0.059 mm (the worst TI), 0.015 ± 0.011 mm (the best DI), and 0.019 ± 0.015 mm (the worst DI) and concluded that digital impressions are a viable alternative to conventional impressions for fabricating frameworks for full arch implant supported prostheses. Unfortunately, as those studies evaluated 3D deviations, it was not possible to directly compare the values from other studies that assessed conventional and digital impressions for full arch implant supported prostheses [10, 26] rather than the gap between the abutment and the prosthesis. According to a recent clinical study, both techniques achieve a satisfactory degree of precision for full arch implant supported prostheses [16]. Conversely, an in vitro study suggested that the digital method was less accurate [10], and a systematic review was unable to establish clinical guidelines on the most precise impression technique due to insufficient high-quality evidence [27]. It is crucial to examine the types of scanners used in studies reporting lower accuracy for digital impressions [10], as more recently developed scanners generally yield better results [14]. Additionally, variations in scan body type and scanner model can produce different outcomes [11].

Although we may have used the same approach of obtaining a single value for the overall mismatch of a framework by taking the average of two values, we chose to analyze each side separately to check if there’s any discrepancy on one side over the other which may be related to the acquisition technique. At MUA #35 (whether MUA #35 or #45 screwed), DI (MUA #35 screwed) = 44.59 ± 2.92 μm, DI (MUA #45 screwed) = 110.04 ± 10.26 μm, CI (MUA #35 screwed) = 51.63 ± 16.80 μm, CI (MUA#45 screwed) = 115.32 ± 11.43 μm, the stitching remained minimal where the IOS and tabletop scanner scan began. Thus, no statistically significant difference was found in the mean vertical misfit between the two groups studied at #35 (*P* =.199). Contrariwise, at MUA #45, DI (MUA #35 screwed) = 92.74 ± 15.46 μm, DI (MUA #45 screwed) = 53.73 ± 8.80 μm, CI (MUA #35 screwed) = 114.33 ± 15.91 μm, CI (MUA #45 screwed) = 60.56 ± 4.93 μm, the image stitching escalates as we progress toward the end of scanning the arch the stitching, leading to a significant difference between the DI and CI groups, though still within acceptable misfit levels documented in the literature.

Statistical analysis of the DI intragroup comparison revealed significant differences in the average vertical misfit (*P* <.001) where MUA #45 showed statistically significantly higher average vertical misfit compared with MUA #42, #32, and #35 whether (MUA #35 or #45 is screwed). This discrepancy suggests that the large area to be scanned increases the risk of minor deviations accumulating during the formation of three-dimensional images and that the IOS might face challenges in accurately capturing the arch due to its shape. Thus, employing the correct scanning strategy is crucial to mitigate this issue. Consequently, no definitive conclusion can be drawn regarding the superiority of one technique over the other, as various factors contribute to the differing results. Nonetheless, recent studies tend to show improved outcomes, likely due to the use of more advanced scanners. Selecting the optimal impression technique is a crucial decision for ensuring the success and longevity of prosthetic rehabilitation.

The study was limited to a specific IOS Medit i-700; other available IOSs should also be evaluated for their accuracy and predicted errors. In addition, difficulties with full arch digital implant scanning include greater inter-implant distances and edentulous segments lacking in geometric structure, resulting in image superimposition inaccuracies and system differentiation between identical implant scan bodies. Another possible limitation of the current study is the presence of variables such as oral tissues, saliva, blood contamination, and patient movements that might influence the outcome data. The fact that the investigation was carried out at room temperature (23 °C), as opposed to oral temperature (37 °C), is another limitation. This may have produced more precise impressions than those used in clinical practice because the thermal contraction of the conventional impression materials was not considered. As digital technology forges ahead, it is expected that both hardware and software systems will improve, leading to enhanced digital impressions for cases with full arch implants. To draw more firm results, future clinical research must thoroughly compare the various impression techniques and address the numerous influencing variables.

## Conclusion

The following conclusions were drawn based on the findings of this in vitro study:

1. Although the CI and DI groups show a significant difference, the misfit levels remain within the ranges considered acceptable in the literature.
2. Digital impression techniques showed promise as potential alternatives to conventional methods for achieving a passive fit.
3. There is limited scientific evidence verifying the clinical reliability of VSXE impression material for implant supported prostheses. However, the misfit levels associated with its use remain within an acceptable range.

## Data Availability

The datasets used and/or analyzed during the current study are available from the corresponding author upon reasonable request.

## Abbreviations

CI: Conventional impression
VSXE: Vinyl siloxane ether
DI: Digital impression
IOS: Intraoral scanner
CAD-CAM: Computer-aided design and computer-aided manufacturing
MUAs: Multi-unit abutments
